# Temporal Lobe Cystic Collection and Associated Oedema: A Rare Complication of Translabyrinthine Resection of Vestibular Schwannoma

**DOI:** 10.7759/cureus.2217

**Published:** 2018-02-21

**Authors:** Abdurrahman Raeiq

**Affiliations:** 1 Neurosurgery, Sir Charles Gairdner Hospital, Perth, Australia

**Keywords:** translabyrinthine, temporal lobe, oedema, cystic collection, complication, vestibular schwannoma

## Abstract

Vestibular schwannomas (VS) are benign tumours arising from the vestibulocochlear nerve. Among the management options available, surgical resection is often considered, especially if lesions are enlarging or symptomatic. Translabyrinthine (TL) resection of a vestibular schwannoma is one of three basic approaches available to the surgeon. Complications generally associated with this approach can include cerebrospinal fluid (CSF) leak, hearing and balance issues, infection, and facial nerve palsies. We present the case of a patient with a previously unreported type of complication: that of cystic CSF collection within the temporal lobe and associated oedema.

## Introduction

Vestibular schwannomas (VS) are benign tumours that develop from the vestibular component of the eighth cranial nerve (VIII) [1]. Management options include observation with serial imaging, radiotherapy, gross total resection, and subtotal resection with planned radiation therapy [1]. The decision to pursue surgery is based on size, location, symptoms, and experience of the surgeon. Once surgery is decided there are three basic approaches: translabyrinthine (TL), retrosigmoid, and through the middle cranial fossa. The TL approach is often considered in patients with hearing loss or impairment allowing a safe corridor to the posterior fossa with minimum brain retraction and a favourable position of the vestibular component of the vestibular nerve [1]. Complications of TL resection of VS have been reported to include meningitis, cerebrospinal fluid (CSF) leak, headache, balance disturbance, tinnitus, and facial nerve (FN) palsies [2]. There have not been any prior reports of cystic collections within the temporal lobe postoperatively following TL resection of VS; however, there has been an isolated report of a subdural hygroma (SH) [3] and some reports of temporal lobe oedema [3,4]. We present the case of a cystic collection within the temporal lobe and associated oedema, after routine TL resection of a vestibular schwannoma, presenting with neurological deterioration in the postoperative period—a previously undocumented complication in the medical literature.

## Case presentation

**Figure 1:**
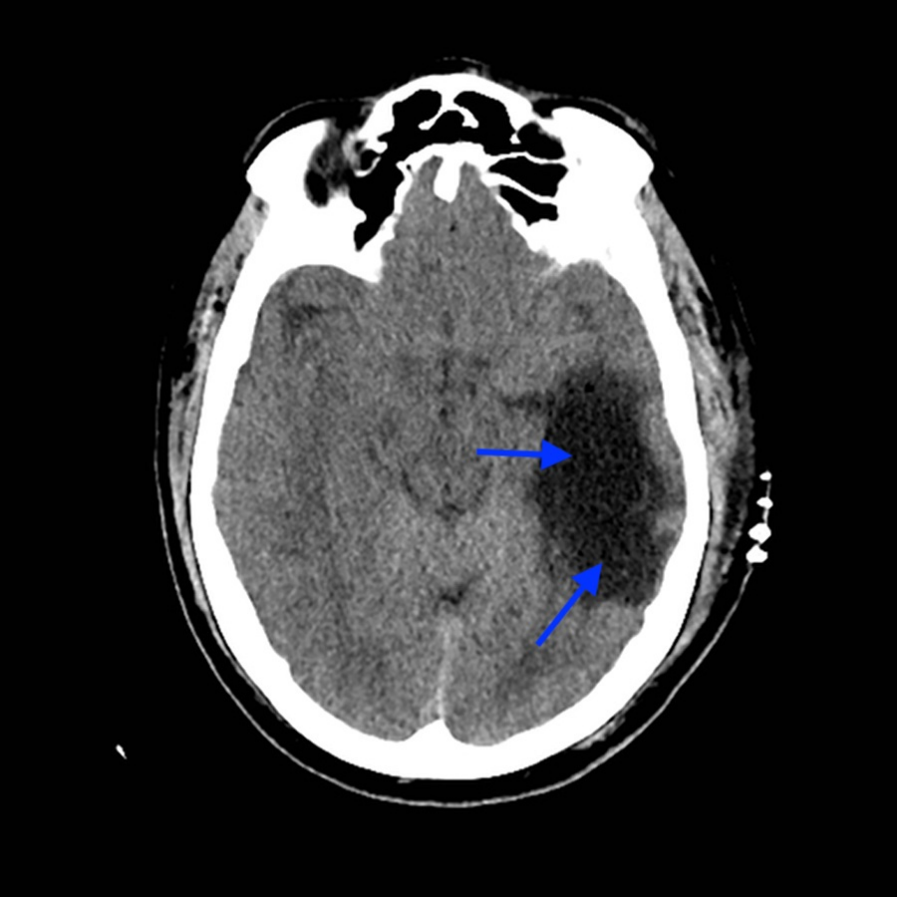
Axial unenhanced CT of the head with large lucency (blue arrows) in the left temporal lobe. CT - computed tomography

**Figure 2:**
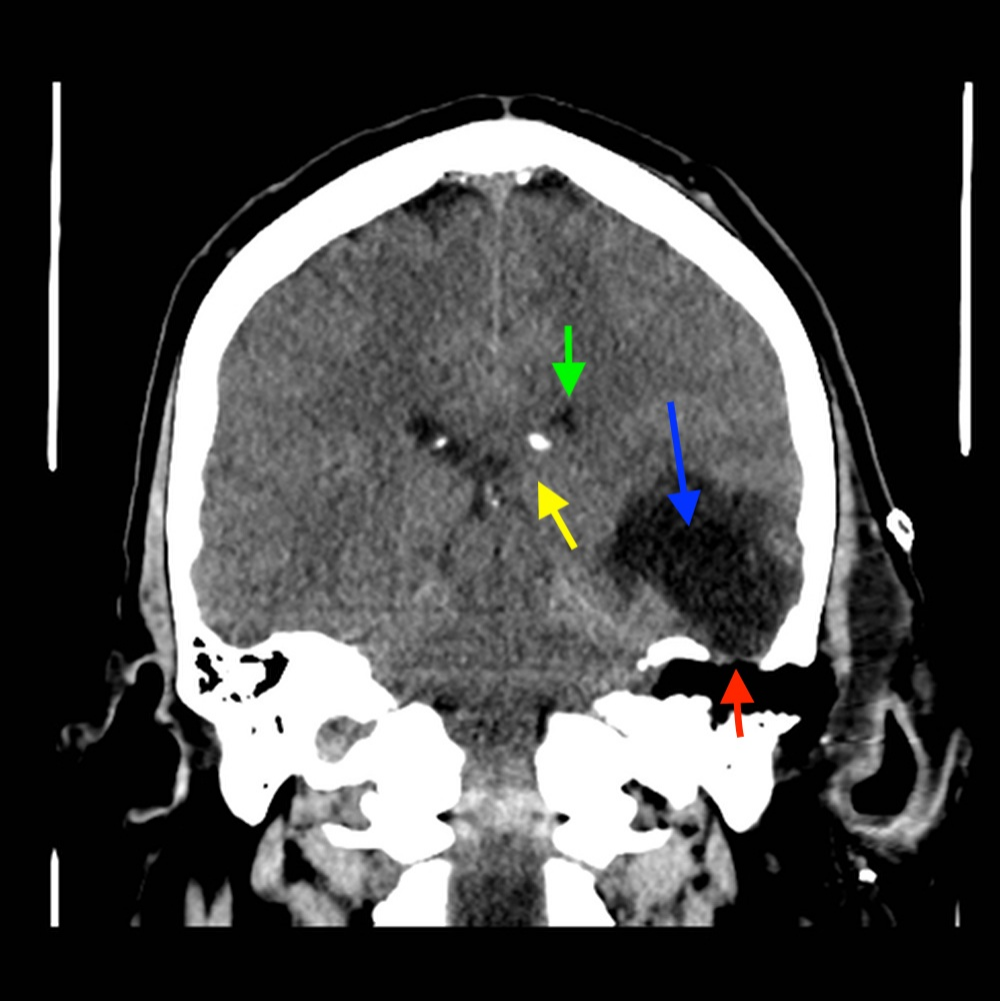
Coronal unenhanced CT of the head at the level of EAM: large lucency left temporal lobe (blue arrow), tegmen tympani defect (red arrow), mass effect on left lateral ventricle (green arrow) and on medial temporal lobe (yellow arrow). CT - computed tomography, EAM - external acoustic meatus

**Figure 3:**
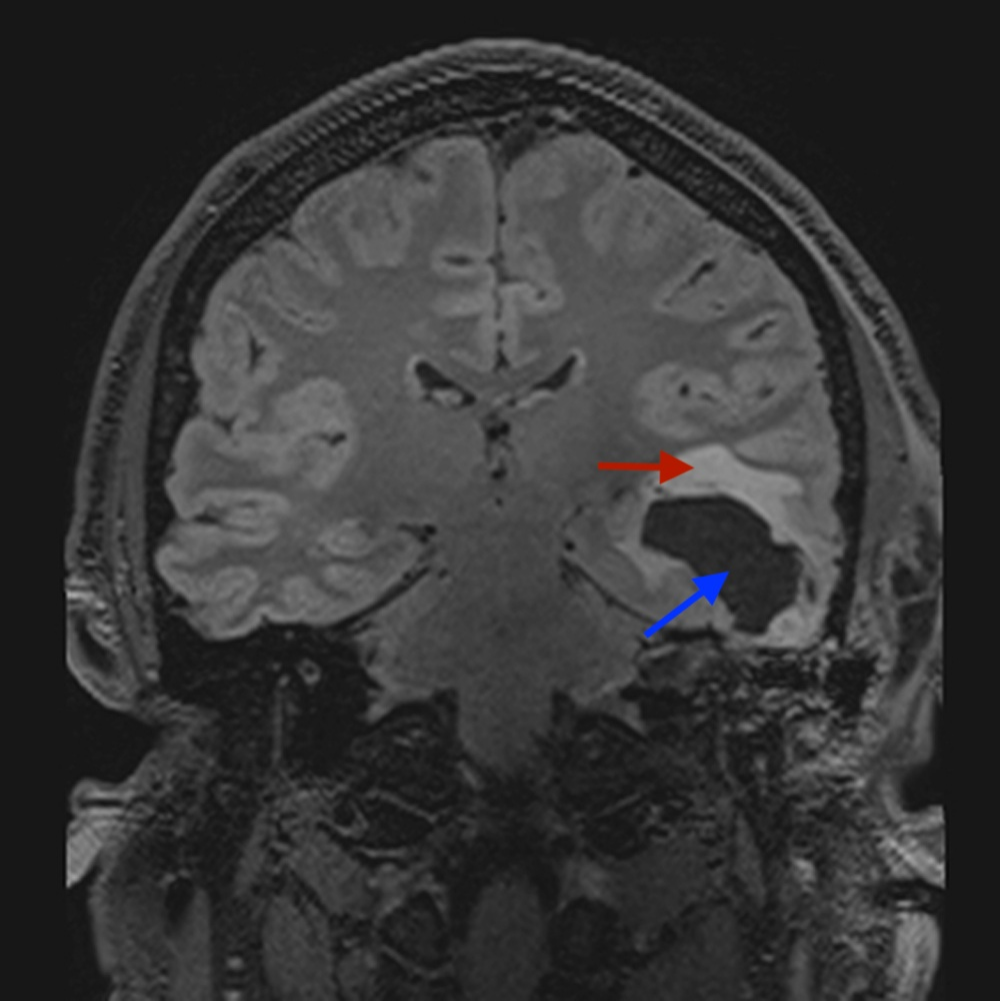
Coronal unenhanced T2 fat suppressed MRI of the brain: lucency in the temporal lobe (blue arrow) and associated surrounding oedema (red arrow). MRI - magnetic resonance imaging

**Figure 4:**
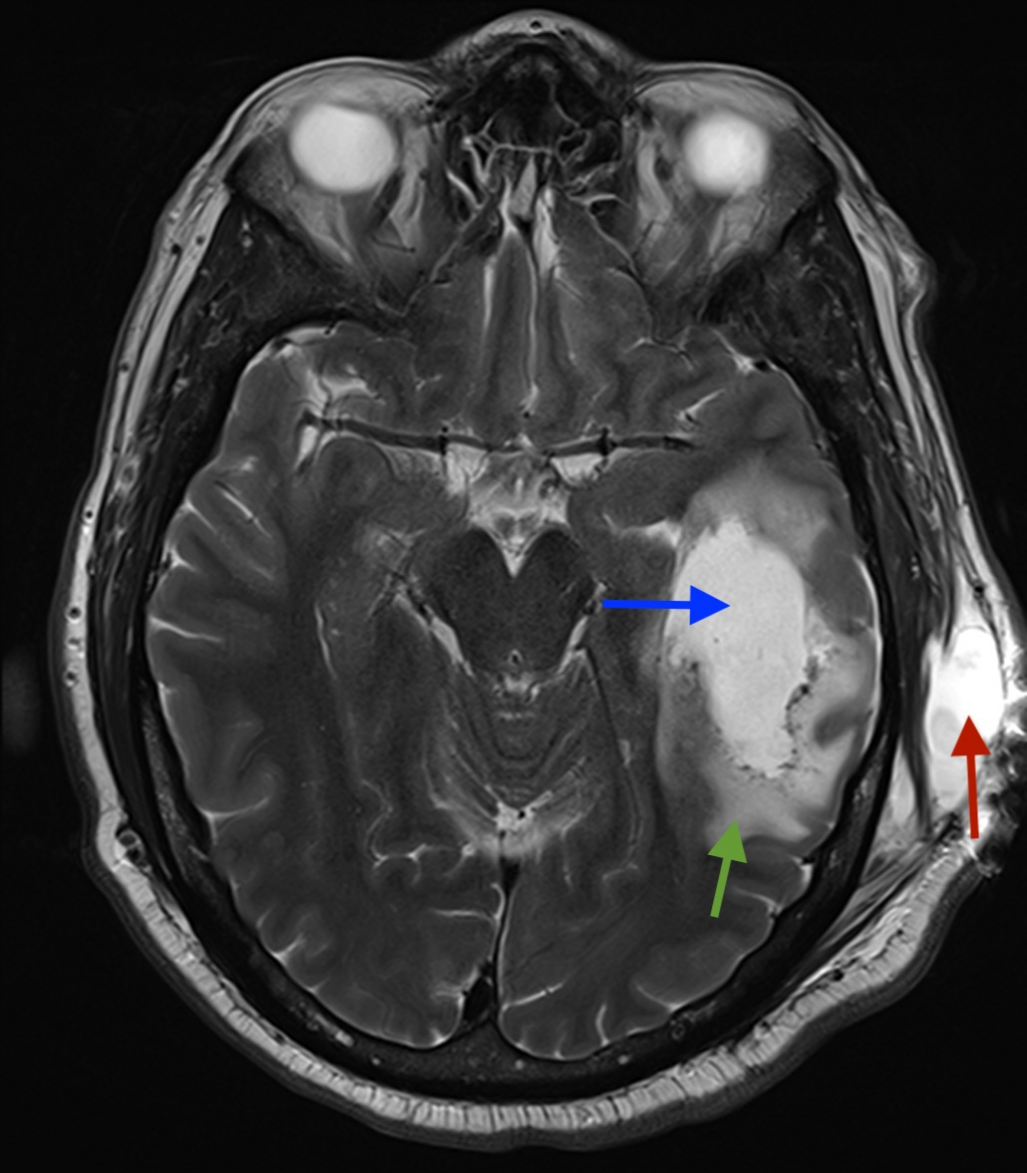
Axial unenhanced T2 MRI of the brain: lucency in temporal lobe (blue arrow) and associated surrounding oedema (green arrow), pseudomeningocoele (red arrow) in subcutaneous tissues on the left side.

A 49-year-old male was admitted electively for a TL resection of a vestibular schwannoma. He had symptoms of a left sensorineural hearing loss and pain in the distribution of the trigeminal nerve, for seven months, prior to the diagnosis of a left intracanalicular vestibular schwannoma. The patient underwent an uneventful procedure. On the fourth day after the operation he became confused with mild dysphasia. A computed tomography (CT) of his brain showed a hypodense collection in the left temporal lobe (Figures 1-2). Further investigation with magnetic resonance imaging (MRI) of his brain demonstrated a fluid collection within the substance of the left temporal lobe and significant oedema of the surrounding temporal lobe with mass effect, but there was no evidence of haemorrhage or abscess formation (Figures 3-4). In addition there was evidence of pseudomeningocele formation in the subcutaneous tissues adjacent to the previous wound. The patient showed further worsening of his confusion and speech disturbance and the decision was made to proceed to the operating theatre.

**Figure 5:**
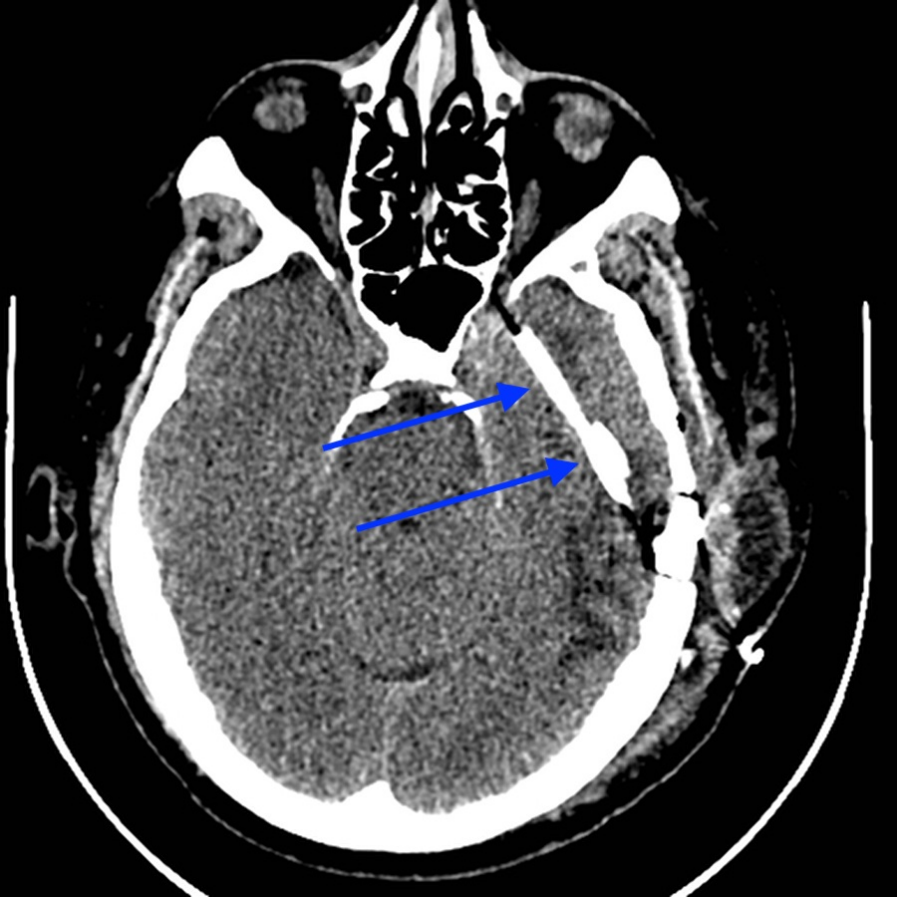
Postoperative unenhanced axial CT of the head: shunt (blue arrows) in situ. CT - computed tomography

**Figure 6:**
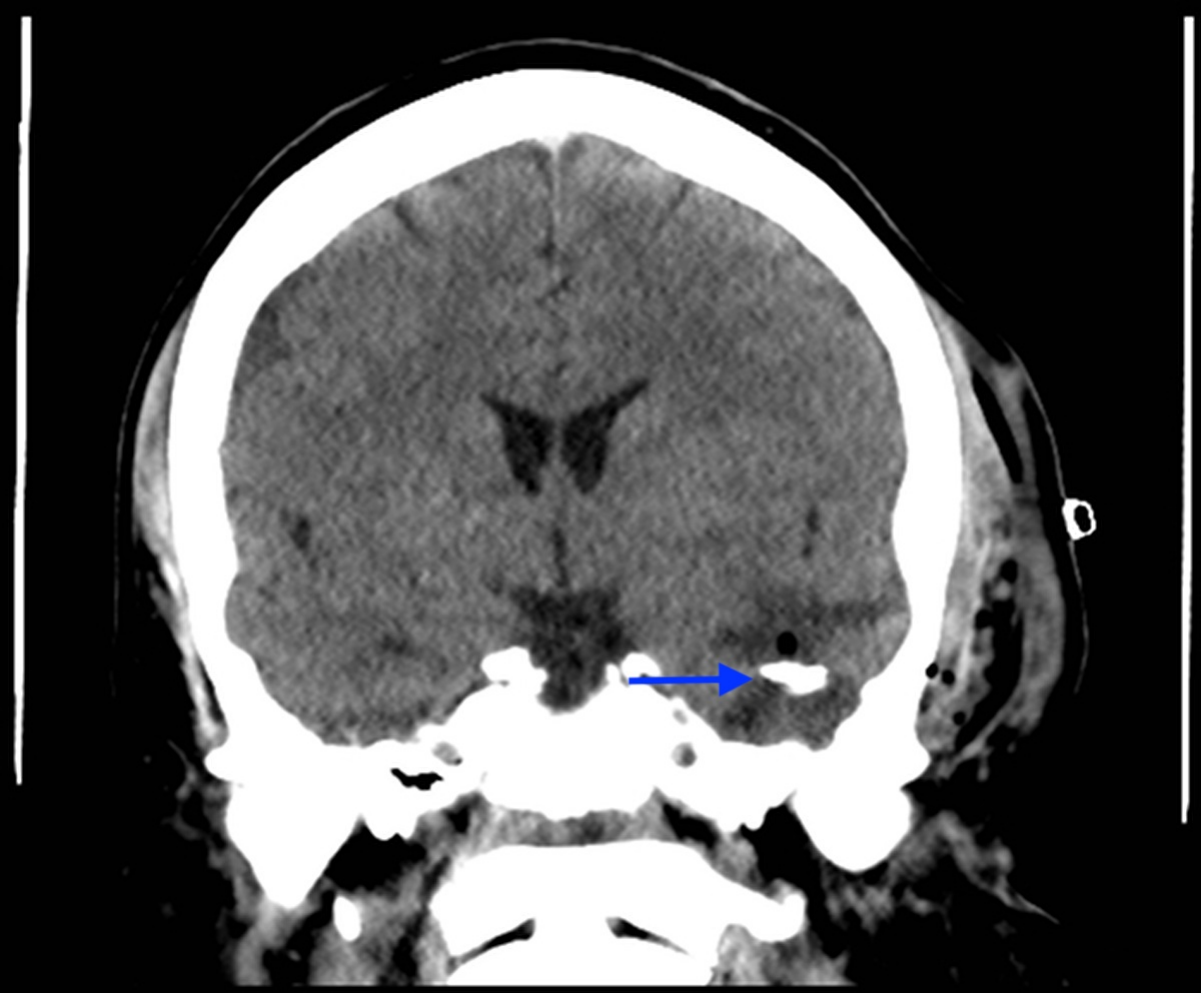
Postoperative unenhanced coronal CT of the head: shunt tubing (blue arrow) sitting in previous cystic cavity, which is now collapsed around the tube. CT - computed tomography

**Figure 7:**
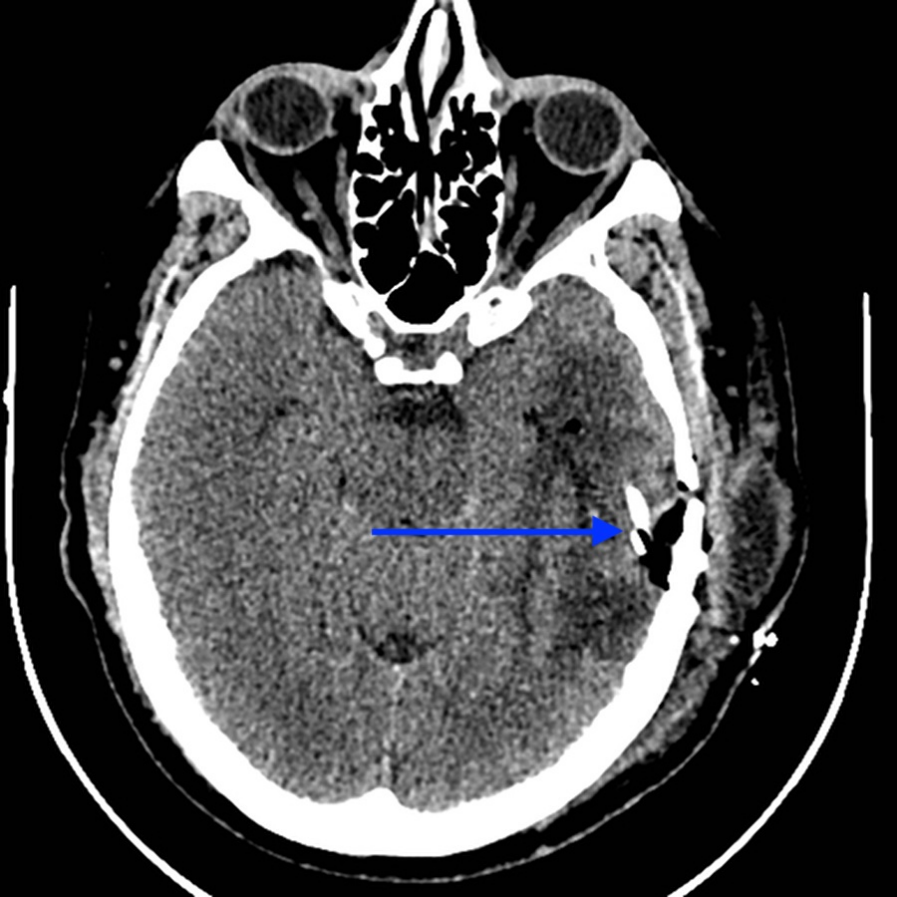
Postoperative unenhanced axial CT of the head: end of shunt tube (blue arrow) in subdural space. CT - computed tomography

At operation the pseudomeningocele was evacuated; a 2x2 cm craniotomy was performed over the squamous temporal bone. After elevation of the bone flap, a small dural tear was noted over the inferior temporal lobe and the temporal lobe was bulging against the dural margins. Bipolar cortectomy was performed and the cavity identified on MRI was entered with egress of clear CSF under high pressure. The temporal lobe immediately appeared relaxed and was seen to be pulsating normally. A cystosubdural shunt (CSS) was fashioned from two 5 cm pieces of a 1.9 mm diameter external ventricular drain. Both were inserted into the cystic cavity with the other ends placed in a subdural pocket and secured to dura with sutures. Haemostasis was achieved and copious washout of the surgical site was followed by layered closure with absorbable sutures and nylon and staples to the skin. After reversal of anaesthesia and extubation, the patient showed some improvement in his confusion. A CT scan of the head 24 hours postoperatively showed resolution of the cystic collection and improvement of the mass effect (Figures 5-7). He was continued on anti-seizure medications prophylactically for seven days postoperatively. Over the next few days his confusion and speech improved rapidly and he was referred on for further rehabilitation.

## Discussion

Typical complications of TL resection of VS include meningitis, CSF leak, headache, balance disturbance, tinnitus, and FN palsies. Additionally, one can expect hearing loss with the TL approach, often pre-existing and a contributing reason to the decision to operate via this approach [1,2]. Cystic temporal lobe collections of CSF under pressure have never been reported in literature post TL resection of VS. There has been one single published report of an SH and temporal lobe oedema post a TL resection of a vestibular schwannoma [3]. It was postulated that dural injury to the superior petrosal sinus and inadvertent injury to the underlying arachnoid with accumulation of CSF in the subdural space, with an associated one way valve effect, was a possible cause of that presentation. The underlying oedema has previously been linked to progressive venous ischaemia secondary to mechanical pressure on venous structures [3]. Venous infarction has been reported in three cases after superior petrosal sinus resection to facilitate exposure to the vestibular schwannoma and was thought to account for the temporal lobe oedema identified on CT imaging of the brain [5]. However, it was not clear whether venous flow insufficiency was proven. None of these reports, however, explain the complication in this case.

**Figure 8:**
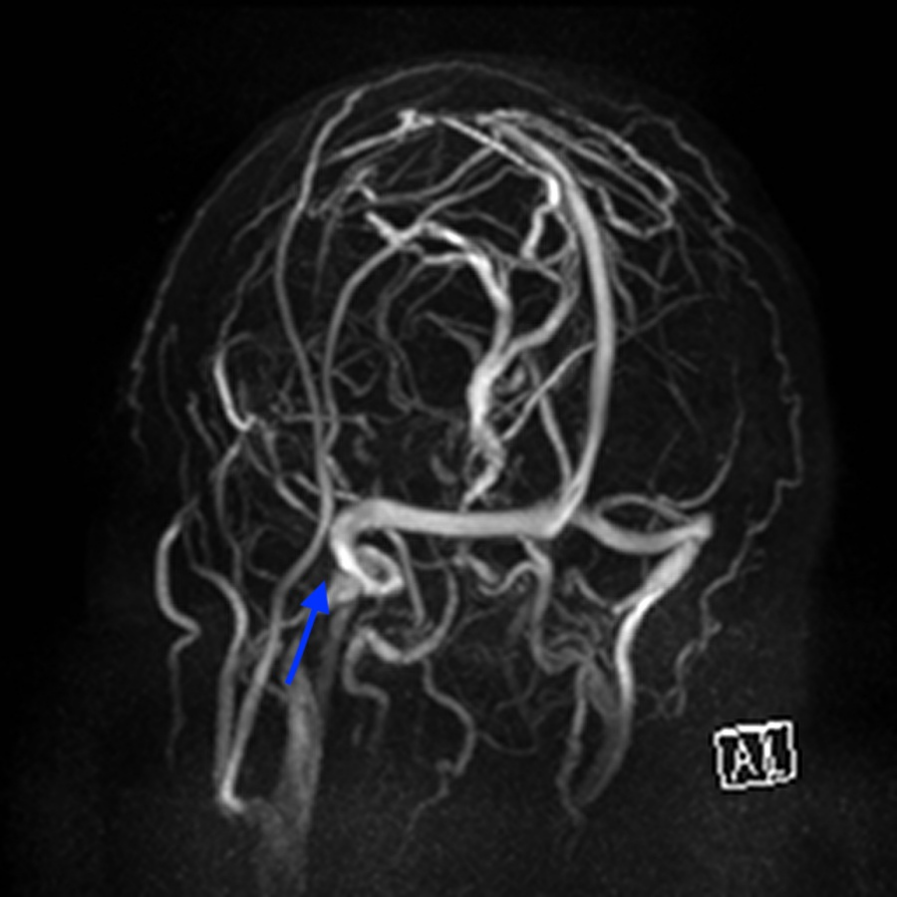
Magnetic resonance venography of the dural venous sinuses. Note the left side is the left side of the picture, no filling or flow defects identified in the area of the operation (blue arrow).

The exact pathophysiology of this lesion is unknown. We postulate that a possible iatrogenic leucotomy was achieved through unrecognised injury to the cortex and overlying dura and arachnoid. The resultant lesion continued due to build up of CSF through a one way valve effect, which would account for the delayed presentation. The isolated temporal lobe oedema was similarly noted compared with previous reports [3,4]. In one series of 84 patients who were routinely scanned 24 hours post resection of a vestibular schwannoma, 14 developed temporal lobe lucency, suggesting oedema, infarction, or ischaemia, and all of these patients had undergone a TL approach for the vestibular schwannoma resection [4]. Thus there appears to be a definite link with this approach and the development of temporal lobe oedema. However, in relation to this case no intraoperative issues were encountered with dural sinus injury. It is possible local coagulation with bipolar cautery on the margins of the dural venous sinuses (DVS) could have precipitated venous ischaemia with subsequent oedema and not have been appreciated at the time of surgery. However, an MRI venography demonstrated normal flow in the DVS (Figure 8). In this case the collection of CSF was under high pressure within the temporal lobe and local mechanical effects could have contributed to venous insufficiency and development of oedema as opposed to iatrogenic injury during time of surgery. The development of the cystic CSF collection, however, can not be explained in the temporal lobe without some mechanical force allowing breach of the cortex and entry of the CSF. The exact nature of this mechanical force is not clear and could be a result of multiple factors, including direct or transmitted drill trauma, tissue damage due to suctioning, and damage to tissues secondary to the pressure of fluid used to wash the cavity. All these factors could have also been present in the context of local venous ischaemia and or infarction and resultant friability of the surrounding brain tissue.

## Conclusions

Cystic CSF collection in the temporal lobe is a previously unreported complication of TL resection of VS. This rare and unusual complication demonstrates significant morbidity to patients. The diagnosis can be suspected on CT, but MRI is useful to rule out venous infarction and abscess. Early treatment is warranted if clinical deterioration occurs, presumably due to increase in size and therefore mass effect of the collection. During surgery the overlying dural and arachnoid planes must be treated with care without undue pressure on underlying structures or repeated trauma to the dura with drilling and or other equipment, which can be transmitted to the cortical tissue and possibly cause inadvertent leucotomy and trapped CSF causing mass effect and venous insufficiency.

This case demonstrated a rare complication of TL of a vestibular schwannoma with its associated features, clinical course, radiological findings, and a novel approach to shunting the cyst to the subdural space to relieve symptoms. The complication was recognised and with prompt treatment the patient made a good neurological recovery with gradual resolution of the temporal lobe findings and improvement in his speech and confusion.
